# A Comparative Assessment of the Efficiency of Orthodontic Treatment With and Without Photobiomodulation During Mandibular Decrowding in Young Subjects: A Single-Center, Single-Blind Randomized Controlled Trial

**DOI:** 10.1089/photob.2019.4747

**Published:** 2020-05-19

**Authors:** Antonino Lo Giudice, Riccardo Nucera, Rosalia Leonardi, Alessio Paiusco, Marco Baldoni, Gianluigi Caccianiga

**Affiliations:** ^1^ Section of Orthodontics, Department of Medical-Surgical Specialties, School of Dentistry, University of Catania, Policlinico Universitario “V. Emanuele,” Catania, Italy.; ^2^ Section of Orthodontics, Department of Biomedical and Dental Sciences and Morphofunctional Imaging, School of Dentistry, University of Messina, Policlinico Universitario “G. Martino,” Messina, Italy.; ^3^ Section of Orthodontics, Department of Surgery and Interdisciplinary Medicine, School of Medicine and Surgery, University of Milano-Bicocca, Milan, Italy.

**Keywords:** orthodontic treatment, photobiomodulation, accelerated dental movement

## Abstract

***Objective:*** To assess if photobiomodulation (PBM) improves the efficiency of orthodontic treatment with fixed appliance during the alignment stage.

***Methods:*** Eighty-nine subjects were included in this trial and randomly assigned for treatment with fixed appliance and PBM group or with fixed appliance only (control group). Inclusion criteria were as follows: (1) age between 13 and 30 years, (2) permanent dentition, (3) class I malocclusion, (4) lower 6–6 mild crowding measured on dental cast, (5) no spaces or diastema in the lower arch, (6) no ectopic teeth, (7) nonextractive treatment plan, and (8) no previous orthodontic treatment. PBM was administered in the PBM group every 14 days using the ATP38^®^ (Biotech Dental, Allée de Craponne, Salon de Provence, France) (72 J/cm^2^ of fluency for each session). Dental alignment was assessed by visual inspection, and treatment time was defined in days as T2 (date of assessment of complete dental alignment)–T1 (date of brackets bonding). The number of monthly scheduled appointments was also recorded. All the data underwent statistical analysis for comparison between groups.

***Results:*** Treatment time was significantly shorter (*p* < 0.001) in the PBM group (203 days) compared with the control (260 days). Consequently, control visits (*p* < 0.001) were lower in the PBM group (7) compared with the control group (9).

***Conclusions:*** The present findings would confirm that PBM can be used to enhance the efficiency of orthodontic treatment during dental decrowding.

## Introduction

The duration of the orthodontic treatment is associated with higher risk of enamel demineralization, gingivitis, alveolar bone loss, and root resorption.^1–3^ A significant percentage of subjects, especially adults, still refuse orthodontic treatment since they are concerned about the extended treatment time, which may have a negative impact in daily life, in particular if fixed appliance are used.^4^ Secondary, clinicians would desire to fasten the orthodontic treatment to reduce patients' chair-time, facilitating their business.^5^ Thus, improving the efficiency of orthodontic treatment is a primary concern for both patients and clinicians.^6^

Surgical procedures such as corticotomy^7,8^ and accelerated osteogenic orthodontics^9,10^ have been proposed as effective methods to accelerate orthodontic tooth movement, however, these methods cause more discomfort than conventional treatment and require highly skilled oral surgeons and specific surgical equipment. In this respect, nonsurgical methods for accelerating orthodontic treatment such as the daily use of portable vibration device or electric toothbrush can be considered patient-friendly approaches, however, there is no sufficient evidence of their effectiveness.^11,12^

Photobiostimulation is a noninvasive irradiation procedure that uses a laser light within the red to near-infrared range (wavelengths from 632 to 1064 nm) to provoke a biological reaction. In vitro studies reported that photobiostimulation accelerates cellular turnover by increasing the expression of osteocalcin,^13^ stimulating angiogenesis,^14^ and the availability of mitochondrial ATP.^15,16^ In the orthodontic field, the assumption is that such increased metabolic activity could speed the rate of tooth movement, as confirmed by some clinical studies.^17–19^ In particular, preliminary findings from two pilot studies^20,21^ suggested that photobiostimulation could reduce orthodontic treatment time in subjects affected by mandibular crowding and confirmed the necessity and the clinical validity of performing further prospective trials with wider sample size to provide definitive conclusions.

In this respect, the aim of the present randomized clinical trial was to evaluate if photobiomodulation (PBM) can improve the efficiency of orthodontic treatment, in particular the time to resolve dental crowding in a sample of subjects undergoing a nonextraction orthodontic treatment plan.

## Materials and Methods

This randomized, parallel (1:1), single-operator clinical trial was performed observing the guidelines of the Declaration of Helsinki and was approved by the Ethics Committee of the Faculty of Medicine at the Milano-Bicocca University. Subjects were recruited and treated between September 2016 and July 2019 and signed an appropriate informed consent for the orthodontic treatment; also, patients in the tested group signed a specific consent for the PBM sessions.

### Human subjects

One hundred subjects were recruited from a larger pool of patients (334) seeking orthodontic treatment at an orthodontic private clinic in Bergamo, Italy. A minimum sample size of 80 participants (40 for each group) was considered to obtain 90% power at a 95% confidence interval to demonstrate a difference of 56 days in treatment time between the PBM group and control group, as previously reported.^20^ However, we decided to enroll 100 subjects (50 for each group) counteracting any potential incompleteness of data. The enrollment process was based on the following criteria: (1) age between 13 and 30 years, (2) permanent mandibular dentition, (3) angle class I malocclusion, (4) lower 6–6 mild crowding measured on dental cast, (5) no diastema or spaces in the lower arch, (6) no ectopic teeth, (7) no extractions required or intraoral or extraoral auxiliary devices, and (8) no previous orthodontic treatment.

A randomized balanced block protocol using sex and the amount of crowding as stratification factors was created to allocate subjects to receive orthodontic treatment with fixed appliance plus PBM group or with fixed appliance only (control group). The SPSS Statistics software (IBM Corporation, Armonk, New York) was used to generate the allocation sequence. Assignments were enclosed in sequentially numbered, sealed, and opaque envelopes and were unveiled the date of bonding the fixed appliance.

### Intervention

The appliance used in both groups was the Empower self-ligating appliance (American Orthodontics, Sheboygan, WI) with 0.022-in slot and MBT prescription. The arch-wire sequence included 0.014-in thermal NiTi arch-wire followed by 0.016 × 0.022-in and 0.019 × 0.025-in thermal NiTi arch-wires (Thermal-Ti Lite, Form I; American Orthodontics). The arch-wire sequence progressed only if full bracket engagement was easily feasible, that is, without forcing the rectangular wires throughout the bracket system. Control visits were scheduled at intervals of 28 days to check clinical progress and to adjust the appliance, if necessary. Some days of delay for each appointment would be tolerated if important adjustments were not required such as arch-wire replacement and brackets rebonding; however, we decided to use a cutoff of 14 days of collected delay to include/exclude subjects from the final investigation. Bracket bonding and clinical controls (including the adjustment of the appliance and arch-wire) were entrusted to the same expert operator (G.C.).

**FIG. 1.**
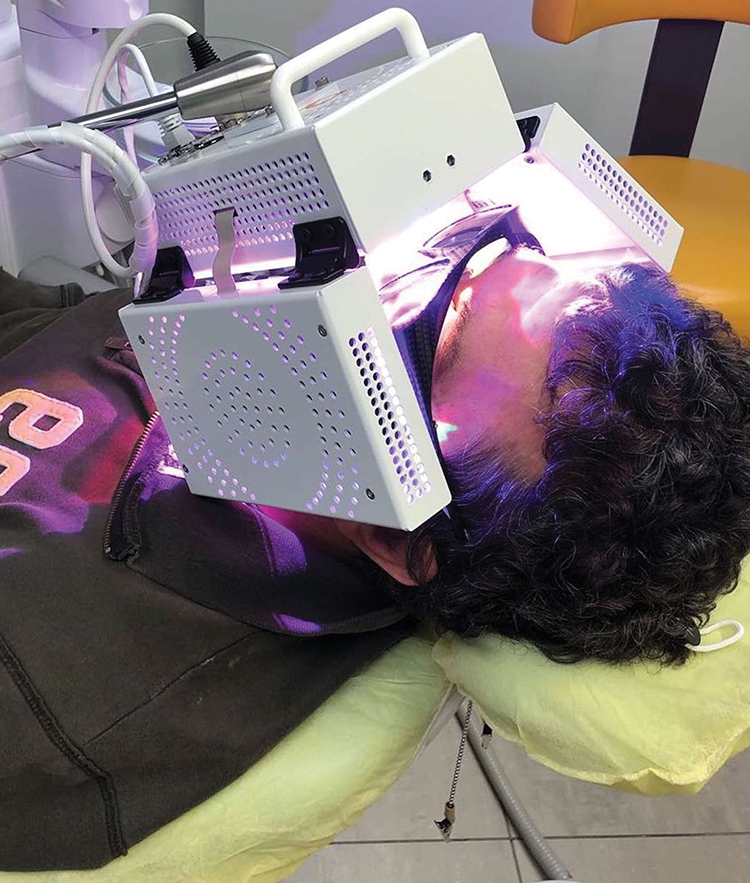
The ATP38^®^ (Biotech Dental, Allée de Craponne, Salon de Provence, France) was used in this study to perform PBM. See the three panels placed at a 4 cm distance from the patient's cheeks (lateral panels) and lips (frontal panel). PBM, photobiomodulation.

PBM was administered to the PBM group using the ATP38^®^ (Biotech Dental, Allée de Craponne, Salon de Provence, France). This device features a multi-panel system emitting cold polychromatic lights with a combination of wavelengths from 450 to 835 nm depending on the field of action, that is, the part treated and the therapeutic indication (healing, anti-inflammatory, and analgesic effect) (Fig. 1). For the purpose of the present investigation, the biostimulation module was selected according to the manufacturer's instructions; this module provided 6 min of irradiation producing 48 J/cm^2^ of fluency, calculated as the sum of the fluency produced by each light source (16 J/cm^2^) multiplied for the three active panels (16 J/cm^2^ × 3 = 48 J/cm^2^) (Table 1). These data were based on a fixed distance of 4 cm of the three panels to the patient's cheeks (lateral panels) and lips (frontal panel).

**Table 1.** Photobiostimulation Irradiation Parameters According to the Biostimulation Module of the ATP38 Device

	Cold lights' combination					
	Blue	Green	Amber	Red	Deep red	Infrared
Wavelengths	470 nm	525 nm	590 nm	620 nm	680–760 nm	800–835 nm
Duration (sec)^a^	298/368	363/368	332/368	368/368	320/368	362/368
Fluency (J/cm^2^)^b^	2	2	1	3	4	4
Frequency (Hz)	99	99	99	99	99	99

^a^ Duration is intended as the irradiation time for each cold light source out of the total time of PBM stage, which was 368 sec.

^b^ Total fluency (16 J/cm^2^) is intended as the sum of the fluency produced by each cold light source for each panel. Since the ATP38^®^ device consists of three static panels, the total fluency is 48 J/cm^2^.

Data in table are reported for a single stage of biostimulation module. However, since in this study each PBM session consisted of three repeated stages, total fluency and total duration must be multiplied by 3. Thus, total duration was 18 min and produced 144 J/cm^2^ of fluency.

PBM, photobiomodulation.

Since 48 J/cm^2^ was notably below the fluency range used for photobiomodulated orthodontics,^20–22^ we included three consecutive stages of irradiation in each PBM session, for a total duration of 18 min and 144 J/cm^2^ of fluency administered (i.e., 48 J/cm^2^ × 3 stages). A rest time of 1 min was set between each stage. Each PBM session was performed every 14 days, including the date of bracket bonding, up to the end of the alignment stage. Thus, the fluency administered to the patients was 288 J/cm^2^ per month (144 J/cm^2^ × 2 sessions). Specific irradiation parameters such as wavelength, duration, fluency, and frequency are reported in Table 1.

### Assessment of dental alignment treatment time

A digital caliper (Absolute Digimatic IP67; Mitutoyo Europe GMBH) was used to quantify the Little's irregularity index in the lower arch (6–6) on the pretreatment dental casts, and all measurements were reported on a spreadsheet. Twenty dental casts were randomly selected and remeasured 4 weeks later. A paired sample *t*-test was applied to the first and second measurements and no differences were found. All measurements were entrusted to one calibrated operator (A.P.).

The assessment of dental alignment (T2) was based on the visual examination of correction of the 11 mandibular interproximal contacts. In this respect, the date of appliance bonding (T1) and the date when complete resolution of crowding was established (T2) were recorded, and alignment treatment time was defined in days as T2–T1. These data were recorded on a spreadsheet along with the total number of monthly scheduled appointments and the collected delay (days) for each participant. The assessment of dental alignment and the relative data registration were entrusted to one expert operator (M.B.) who was unaware of whether the subjects being assessed were within the PBM or control groups.

### Statistics

Descriptive statistics were performed to assess the demographic and clinical characteristics of the study sample. Student's *t*-test and chi-square test were used, respectively, for the evaluation of numerical (age, crowding) and categorical (gender) characteristics between the two groups. Student's *t*-test was also used to evaluate the mean appointment delay between both groups.

Normal distribution of the data (days, rate of alignment, and number of appointments) was preliminarily checked using the Shapiro–Wilk test (http://dittami.gmxhome.de/shapiro). Since data had no normal distribution, they were reported as median, maximum, and minimum values. Treatment duration was assessed in both groups using the survival analysis, the log-rank (Mantel–Cox) test. Survival analysis is recommended when the outcomes are prospectively evaluated as the time elapsing (time-to-event data) before an event is experienced (i.e., the alleviation of dental crowding). Also, the Mann–Whitney *U*-test was performed to comparatively evaluate the total number of appointments from T1 to T2 between the PBM and control groups.

## Results

**FIG. 2.**
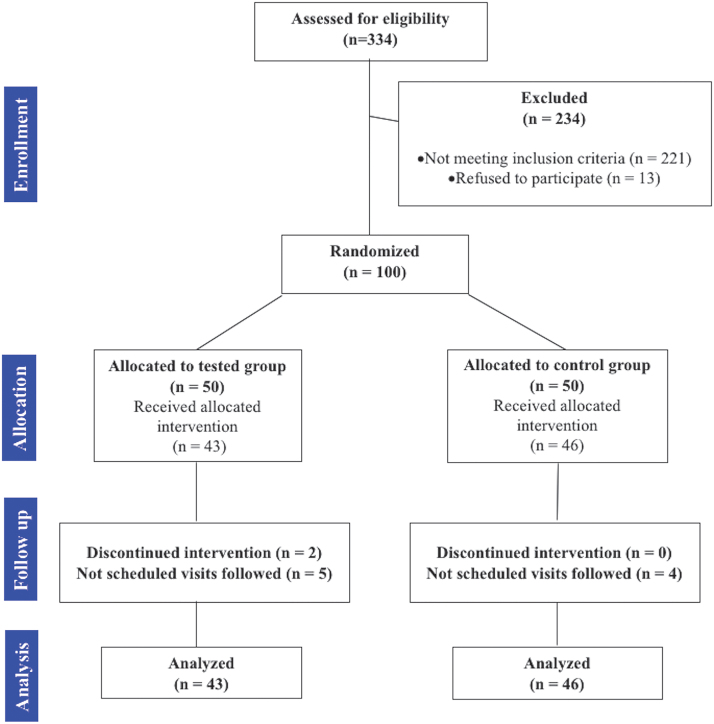
CONSORT flowchart.

From 100 patients enrolled in the present study, 11 were excluded from the final investigation since 2 subjects discontinued the treatment and 9 subjects did not strictly follow the appointment schedule (accumulated appointment delay >14 days or missed important appointment). The final sample size included height—9 subjects (mean age 18.4), including 50 females and 39 males, were finally enrolled. The CONSORT flowchart is reported in Fig. 2. The baseline demographic and clinical characteristics of the study sample, including group division, are shown in Table 2. No differences were found between the two groups for age, sex, amount of crowding, and appointment delay. Thus, the findings of the present study could not be affected by baseline differences between the PBM and control groups.

**Table 2.** Demography, Clinical Characteristics, and Descriptive Statistics of the Sample of the Study

Sample characteristics	Total (*n* = 89)	SD	Control (*n* = 46)	SD	PBM (*n* = 43)	SD	Significance^a^
	Mean or %		Mean or %		Mean or %		
Age (years)	18.42	2.82	17.86	3.79	19.02	4.16	NS
Sex, *n* (%)							
Male	39 (43.82)		19 (41.30)		20 (46.51)		NS
Female	50 (56.17)		27 (58.69)		23 (53.89)		
Crowding (mm)	7.13	1.28	6.89	1.33	7.38	1.21	NS
Appointment delay (days)	4.3	3.62	3.93	3.65	4.69	3.6	NS

^a^ Significance for comparison of group means calculated by paired *t-*test or chi-square test.

Control, orthodontic treatment with fixed appliance; NS, not significant; PBM, orthodontic treatment with fixed appliance and photobiomodulation.

**FIG. 3.**
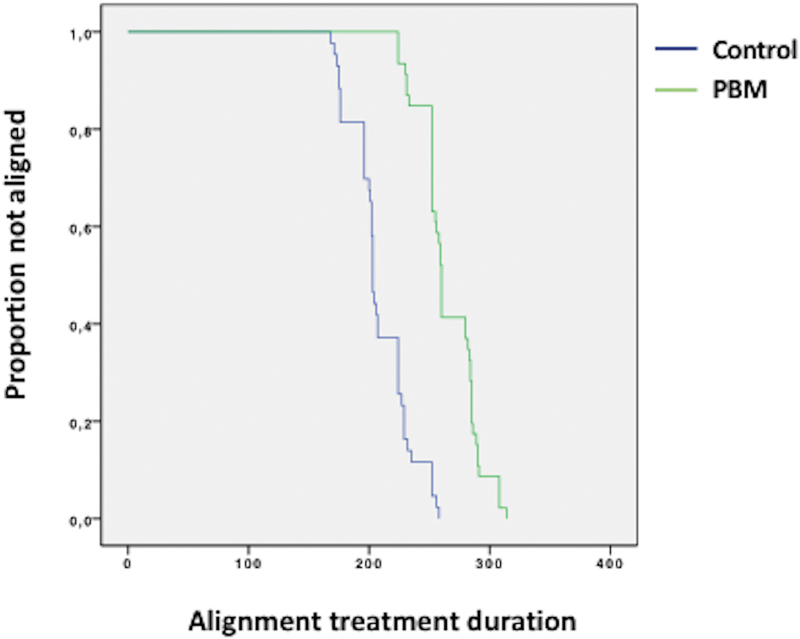
Kaplan–Meier survival curves for the PBM group and control group. The separation between the two curves indicates that the treatment time significantly differed between the PBM group and control group.

According to the survival analysis, the median treatment time was significantly shorter in the PBM group (203 days) compared with the control group (260 days) (*p* < 0.001, Table 3). As a consequence, subjects in the PBM group required less monthly scheduled appointments compared with the controls (seven visits vs. nine visits, as median values), as assessed by the Mann–Whitney *U* test (*p* < 0.001, Table 4). Figure 3 shows the Kaplan–Meier survival curves for the two treatment groups. The space between the two lines indicates that there was difference in treatment duration between the two groups.

**Table 3.** Time to Align Teeth Using Fixed Appliance (Control) and Fixed Appliance Plus Photobiomodulation

	Total	Aligned	Median treatment time	Standard error	95% Confidence interval		Significance^a^
					Lower limit	Upper limit	
Control	46	46	260	0.95	258.13	261.87	*p* < 0.001
PBM	43	43	203	1.09	200.86	205.13	

^a^ *p* Value based on log-rank (Mantel–Cox) test for equality of survivor functions.

**Table 4.** Number of Control Visits for Patients Treated with Fixed Appliance (Control) and Fixed Appliance Plus Photobiomodulation

	Total	Median	Minimum	Maximum	95% Confidence interval	Significance^a^
Control	46	9	8	11	9.1–9.7	*p* < 0.001
PBM	43	7	6	9	6.7–7.2	

^a^ *p* Value based on Mann–Whitney *U* test.

## Discussion

In the present randomized clinical trial, we investigated the efficiency of orthodontic treatment with and without PBM, in particular focusing on the alignment stage. To perform the PBM sessions, we used the ATP38 device, whose effectiveness was never investigated in the dental/orthodontic field.

According to our findings, the median time necessary to resolve lower dental crowding was significantly shorter (*p* < 0.001, Table 3) in the PBM group (203 days) than in the control (260 days). Since potential confounding variables were equally distributed between the two groups (Table 2), our results suggest that PBM increases the efficiency of orthodontic treatment with fixed appliance, in accordance with previous studies.^20–22^

These findings can be explained considering that PBM can create a favorable environment for tooth movement as two types of host responses occur in the irradiated tissues. First, PBM increases the production of mitochondrial ATP, by upregulating the cytochrome c oxidase,^15,23–25^ and promotes cellular viability and the expression of osteocalcin in the tension areas of periodontal ligament (PDL).^13^ This increased metabolic activity accelerates cellular turnover (osteoclast, osteoblast, and fibroblasts)^22,26^ and the production of cytokines involved in the bone remodeling, mainly the IL-1b.^27^ Second, PBM activates the receptor of nuclear factor kappa B (RANK) and the macrophage-colony stimulating factor along with its receptor (c-fms) that, respectively, seem to play a role in the expedition of dental movement.^28^ Further, it seems that PBM may stimulate angiogenesis,^14^ which is also involved in the bone remodeling process, however, further in vitro and in vivo studies are required to deeply elucidate this aspect.

The range of effects of PBM are dependent on light settings, in particular the energy density and the wavelength.^29–31^ The red and near-infrared regions of the spectrum represent the most effective ranges of irradiation, since they thoroughly penetrate into the living tissues to induce cell proliferation and differentiation without overheating the tissues.^8,12,22,23^ In vitro and in vivo studies also suggested that irradiation dose could influence the rate of orthodontic movement. In particular, the effective dosage that was proven to accelerate canine retraction during space closure mechanics is between 150 and 200 J/cm^2^ per month^17,32,33^ and for dental alignment approximately between 260 and 336 J/cm^2^ per month^21,22^ (in these two studies data are reported as daily administration, i.e., 9.3 and 12 J/cm^2^).

Our protocol included two sessions of PBM per month, which produced a total monthly energy density of 288 J/cm^2^, which is in the range of previous studies.^21,22^ A previous randomized pilot study^20^ reported that mandibular dental alignment was expedited using only 150 J/cm^2^ of energy density, however, authors used a diode laser with a wave optical fiber irradiating selectively only the mandibular arch. Conversely, in the present study and in the study of Nahas et al.,^22^ PBM was simultaneously administered in both arches via an extraoral device and in the study of Shaughnessy et al. using an intraoral device.^21^ Thus, it could be postulated that when both maxillary and mandibular arches are irradiated, higher fluency is required since part of the irradiation may be absorbed by the opposite arch (maxillary arch in this case). Nevertheless, the effective dosage at which the target tissues are exposed can be only approximated since different amounts of energy density can be lost through penetration of facial structures,^3^ especially if extraoral devices are used as in the present study.

The alignment treatment time in the PBM group (203 days, as median value) was notably longer than that reported in the study of Shaughnessy et al.^21^ (48 days) and Nahas et al.^22^ (68.3). Both studies,^21,22^ however, limited the assessment of treatment efficiency to the lower anterior teeth (3–3); at the same time, the arch-wire sequence used in the present study included rectangular NiTi wires due to the necessity to correct premolar and molar rotation that is difficult to obtain using only round NiTi arch-wires.^34–38^ Thus, the prospective intrinsic observational time was longer in our investigation due to methodological reasons and, as a consequence, a comparative assessment between the two protocols would be unreliable. This is confirmed even by the longer treatment time in our control sample (260 days) compared with the controls of these studies (104 days^21^ and 87.8^22^). Clinical trials, with parallel arms and standardized orthodontic biomechanics, are required to clarify the appropriate protocol of PBM with regard to the dose, the energy, as well as the number of sessions that can enhance the efficiency of orthodontic treatment.^39^

From a clinical perspective, if the efficiency of orthodontic treatment is enhanced, the exposure of the patients to the risk of undesired effects (cavities, gingivitis, alveolar bone loss, and root resorption) is reduced, which is quite critical. Moreover, from a managerial perspective, reducing treatment time means reducing the total number of appointments and the chair-time, which, in turn, facilitates business.^5^ In the present study, subjects in the PBS had less routine visits compared with the control (7 vs. 9), but they underwent a 24-min in-office session of PBM (Table 1) twice a month, which increased the total number of appointments and chair-time during the treatment.

As a general consideration, the worth of the time reserved for patients depends on the owner's vision of its dental practice. If the goal is to increase the efficiency of the treatment and reduce patient chair-time, home administration of PBS should be preferred using portable device, however, there is no sufficient evidence of the effectiveness.^11,12^ On the contrary, if a clinician/owner considers chair-time as an added value to improve the patients' experience in the dental clinic, in-office administration can be appropriate for this demand. However, a deep evaluation of chair-side time involved in the orthodontic treatment with and without PBM was beyond the aim of the present study.

One of the advantages of using a static device to perform PBM such as the ATP38 is that the session is operator free; this somehow can enhance the standardization of the dosage administered since the operator error is eliminated. However, considering the high costs, it seems more reasonable to use handpiece devices for low-level laser therapy since they were found to improve the patients' experience by reducing orthodontic treatment time^20^ and pain^40^ and also are less expensive.

### Limitations

Data from the present clinical trial should be taken with some caution since they are limited to the stage of dental alignment and should not be related to the total orthodontic treatment time. In this respect, further prospective clinical trials analyzing the efficiency of orthodontic treatment with and without PBM in different consecutive stages, for example, dental alignment, interarch mechanics (class II or class III elastics) and finishing stages or postextractive dental alignment, space closure mechanics and finishing stages, are required to provide more information about the clinical usefulness of PBM in orthodontics.

## Conclusions and Summary

PBM significantly reduces treatment time duration during dental alignment. Considering the lack of side effects, clinicians can refer to PBM to enhance the efficiency of orthodontic treatment in routine clinical practice. However, further clinical trials are required to assess the appropriate protocol of PBM with regard to the pertinent dosage; meanwhile, in vitro and in vivo studies are still necessary to better elucidate the biochemical conditions underlying the positive effects on bone remodeling during the application of orthodontic forces.

## References

[1] Roscoe MG, Meira JB, Cattaneo PM Association of orthodontic force system and root resorption: a systematic review. Am J Orthod Dentofacial Orthop 2015;147:610–626 25919107 10.1016/j.ajodo.2014.12.026

[2] Tanner AC, Sonis AL, LifHolgerson P, et al. White-spot lesions and gingivitis microbiotas in orthodontic patients. J Dent Res 2012;91:853–858 22837552 10.1177/0022034512455031 PMC3420397

[3] Limpanichkul W, Godfrey K, Srisuk N, Rattanayatikul C Effects of low-level laser therapy on the rate of orthodontic tooth movement. Orthod Craniofac Res 2006;9:38–43 16420273 10.1111/j.1601-6343.2006.00338.x

[4] Yassir YA, McIntyre GT, Bearn DR The impact of labial fixed appliance orthodontic treatment on patient expectation, experience, and satisfaction: an overview of systematic reviews. Eur J Orthod 2019. [Epub ahead of print]; DOI: 10.1093/ejo/cjz043 31147683

[5] Buschang PH, Shaw SG, Ross M, Crosby D, Campbell PM Comparative time efficiency of aligner therapy and conventional edgewise braces. Angle Orthod 2014;84:391–396 24749702 10.2319/062113-466 PMC8667515

[6] Skidmore KJ, Brook KJ, Thomson WM, Harding WJ Factors influencing treatment time in orthodontic patients. Am J Orthod Dentofacial Orthop 2006;129:230–238 16473715 10.1016/j.ajodo.2005.10.003

[7] Gibreal O, Hajeer MY, Brad B Efficacy of piezocision-based flapless corticotomy in the orthodontic correction of severely crowded lower anterior teeth: a randomized controlled trial. Eur J Orthod 2019;41:188–195 29931294 10.1093/ejo/cjy042

[8] Caccianiga G, Lo Giudice A, Paiusco A, et al. Maxillary orthodontic expansion assisted by unilateral alveolar corticotomy and low-level laser therapy: a novel approach for correction of posterior unilateral cross-bite in adults. J Lasers Med Sci 2019;10:225–229 31749950 10.15171/jlms.2019.36 PMC6817800

[9] Dab S, Chen K, Flores-Mir C Short- and long-term potential effects of accelerated osteogenic orthodontic treatment: a systematic review and meta-analysis. Orthod Craniofac Res 2019;22:61–68 30884158 10.1111/ocr.12272

[10] Kamal AT, Malik DES, Fida M, Sukhia RH Does periodontally accelerated osteogenic orthodontics improve orthodontic treatment outcome? A systematic review and meta-analysis. Int Orthod 2019;17:193–201 30979612 10.1016/j.ortho.2019.03.006

[11] Katchooi M, Cohanim B, Tai S, Bayirli B, Spiekerman C, Huang G Effect of supplemental vibration on orthodontic treatment with aligners: a randomized trial. Am J Orthod Dentofacial Orthop 2018;153:336–346 29501108 10.1016/j.ajodo.2017.10.017

[12] El-Angbawi A, McIntyre GT, Fleming PS, Bearn DR Non-surgical adjunctive interventions for accelerating tooth movement in patients undergoing fixed orthodontic treatment. Cochrane Database Syst Rev 2015:CD010887 26576758 10.1002/14651858.CD010887.pub2 PMC6464986

[13] Huang TH, Liu SL, Chen CL, Shie MY, Kao CT Low-level laser effects on simulated orthodontic tension side periodontal ligament cells. Photomed Laser Surg 2013;31:72–77 23327633 10.1089/pho.2012.3359 PMC3565557

[14] Eells JT, Henry MM, Summerfelt P, et al. Therapeutic photobiomodulation for methanol-induced retinal toxicity. Proc Natl Acad Sci U S A 2003;100:3439–3444 12626762 10.1073/pnas.0534746100 PMC152311

[15] Oron U, Ilic S, De Taboada L, Streeter J Ga-As (808 nm) laser irradiation enhances ATP production in human neuronal cells in culture. Photomed Laser Surg 2007;25:180–182 17603858 10.1089/pho.2007.2064

[16] Paduano F, Marrelli M, Alom N, et al. Decellularized bone extracellular matrix and human dental pulp stem cells as a construct for bone regeneration. J Biomater Sci Polym Ed 2017;28:730–748 28285576 10.1080/09205063.2017.1301770

[17] Sousa MV, Scanavini MA, Sannomiya EK, Velasco LG, Angelieri F Influence of low-level laser on the speed of orthodontic movement. Photomed Laser Surg 2011;29:191–196 21254890 10.1089/pho.2009.2652

[18] Doshi-Mehta G, Bhad-Patil WA Efficacy of low-intensity laser therapy in reducing treatment time and orthodontic pain: a clinical investigation. Am J Orthod Dentofacial Orthop 2012;141:289–297 22381489 10.1016/j.ajodo.2011.09.009

[19] Caccianiga G, Crestale C, Cozzani M, et al. Low-level laser therapy and invisible removal aligners. J Biol Regul Homeost Agents 2016;30(2 Suppl 1):107–113 27469556

[20] Caccianiga G, Paiusco A, Perillo L, et al. Does low-level laser therapy enhance the efficiency of orthodontic dental alignment? Results from a randomized pilot study. Photomed Laser Surg 2017;35:421–426 28253073 10.1089/pho.2016.4215

[21] Shaughnessy T, Kantarci A, Kau CH, Skrenes D, Skrenes S, Ma D Intraoral photobiomodulation-induced orthodontic tooth alignment: a preliminary study. BMC Oral Health 2016;16:3 26762247 10.1186/s12903-015-0159-7 PMC4711021

[22] Nahas AZ, Samara SA, Rastegar-Lari TA Decrowding of lower anterior segment with and without photobiomodulation: a single center, randomized clinical trial. Lasers Med Sci 2017;32:129–135 27761668 10.1007/s10103-016-2094-5

[23] Tatullo M, Marrelli M, Scacco S, et al. Relationship between oxidative stress and “burning mouth syndrome” in female patients: a scientific hypothesis. Eur Rev Med Pharmacol Sci 2012;16:1218–1221 23047505

[24] Altan BA, Sokucu O, Ozkut MM, Inan S Metrical and histological investigation of the effects of low-level laser therapy on orthodontic tooth movement. Lasers Med Sci 2012;27:131–140 21038101 10.1007/s10103-010-0853-2

[25] Heo JC, Park JA, Kim DK, Lee JH Photobiomodulation (660 nm) therapy reduces oxidative stress and induces BDNF expression in the hippocampus. Sci Rep 2019;9:10114 31300736 10.1038/s41598-019-46490-4 PMC6625994

[26] Goulart CS, Nouer PR, Mouramartins L, Garbin IU, de Fátima Zanirato Lizarelli R Photoradiation and orthodontic movement: experimental study with canines. Photomed Laser Surg 2006;24:192–196 16706698 10.1089/pho.2006.24.192

[27] Varella AM, Revankar AV, Patil AK Low-level laser therapy increases interleukin-1β in gingival crevicular fluid and enhances the rate of orthodontic tooth movement. Am J Orthod Dentofacial Orthop 2018;154:535–544.e5. 30268264 10.1016/j.ajodo.2018.01.012

[28] Fujita S, Yamaguchi M, Utsunomiya T, Yamamoto H, Kasai K Low energy laser stimulates tooth movement velocity via expression of RANK and RANKL. Orthod Craniofac Res 2008;11:143–155 18713151 10.1111/j.1601-6343.2008.00423.x

[29] Ankri R, Lubart R, Taitelbaum H Estimation of the optimal wavelengths for laser-induced wound healing. Lasers Surg Med 2010;42:760–764 20886508 10.1002/lsm.20955

[30] Moore P, Ridgway TD, Higbee RG, Howard EW, Lucroy MD Effect of wavelength on low-intensity laser irradiation-stimulated cell proliferation in vitro. Lasers Surg Med 2005;36:8–12 15662631 10.1002/lsm.20117

[31] Sommer AP, Pinheiro AL, Mester AR, Franke RP, Whelan HT Biostimulatory windows in low-intensity laser activation: lasers, scanners, and NASA's light emitting diode array system. J Clin Laser Med Surg 2001;19:29–33 11547815 10.1089/104454701750066910

[32] Cruz DR, Kohara EK, Ribeiro MS, Wetter NU Effects of low-intensity laser therapy on the orthodontic movement velocity of human teeth: a preliminary study. Lasers Surg Med 2004;35:117–120 15334614 10.1002/lsm.20076

[33] Qamruddin I, Alam MK, Mahroof V, Fida M, Khamis MF, Husein A Effects of low-level laser irradiation on the rate of orthodontic tooth movement and associated pain with self-ligating brackets. Am J Orthod Dentofacial Orthop 2017;152:622–630 29103440 10.1016/j.ajodo.2017.03.023

[34] Lo Giudice A, Nucera R, Matarese G, et al. Analysis of resistance to sliding expressed during first order correction with conventional and self-ligating brackets: an in-vitro study. Int J Clin Exp Med 2016;9:15575–15581

[35] Lo Giudice A, Portelli M, Militi A, et al. Is static friction affected by aging and amount of elastomeric ligatures in orthodontic sliding mechanics? An in-vitro investigation. J Biol Regul Homeost Agents 2018;32(2 Suppl 2):67–73 29542876

[36] Montasser MA, Keilig L, El-Bialy T, Reimann S, Jäger A, Bourauel C Effect of archwire cross-section changes on force levels during complex tooth alignment with conventional and self-ligating brackets. Am J Orthod Dentofacial Orthop 2015;147(4 Suppl):S101–S108 25836341 10.1016/j.ajodo.2014.11.024

[37] Lo Giudice G, Lo Giudice A, Isola G, et al. Evaluation of bond strength and detachment interface distribution of different bracket base designs. Acta Medica Mediterr 2015;31:585

[38] Cordasco G, Lo Giudice A, Militi A, Nucera R, Triolo G, Matarese G In vitro evaluation of resistance to sliding in self-ligating and conventional bracket systems during dental alignment. Korean J Orthod 2012;42:218–224 23112953 10.4041/kjod.2012.42.4.218 PMC3481988

[39] Torri S, Weber JB Influence of low-level laser therapy on the rate of orthodontic movement: a literature review. Photomed Laser Surg 2013;31:411–421 23883115 10.1089/pho.2013.3497

[40] Lo Giudice A, Nucera R, Perillo L, Paiusco A, Caccianiga G Is low-level laser therapy an effective method to alleviate pain induced by active orthodontic alignment archwire? A randomized clinical trial. J Evid Based Dent Pract 2019;19:71–78 30926104 10.1016/j.jebdp.2018.11.001

